# Age-related changes in depressive symptoms in Ghanaian adults

**DOI:** 10.1093/geroni/igaf122.3532

**Published:** 2025-12-31

**Authors:** Nutifafa Dey, Frank Infurna

**Affiliations:** Arizona State University, Tempe, Arizona, United States; Arizona State University, Tempe, Arizona, United States

## Abstract

Depression is a common mental disorder that is among the leading causes of disability worldwide, linked to poor physical health and decreased quality of life trajectories in adulthood. Current evidence from Western contexts suggests depression follows a U-shaped curve trajectory across the lifespan. However, its patterning is unclear in low- and middle-income countries, representing distinct cultural, economic, and societal environments. We address this gap by examining age-related depression changes in Ghanaian middle-aged and older adults using longitudinal data from ISSER-Northwestern-Yale Long Term Ghana Socioeconomic Panel Survey (GSPS). Nonlinear Growth Curve models were applied through the Multilevel Framework to three waves of data from 5337 (observations=14645) participants aged 30 + (baseline: M = 48.67, SD = 13.89). The average depression at age 60 (centering age) was 18.62 and rose by 0.38 units per decade after 60. Depression shows a curvilinear association with age, accelerating at older ages. Trajectories of depressive symptoms across adulthood in Ghana seem to follow the popular U-Shape curve pattern. Age alone explains little within-person depression variability, and more research is needed to examine the effects of other predictors. Intervention should target depression in later life to address increasing mental health issues and promote quality of life.

